# Rockpool Gobies Change Colour for Camouflage

**DOI:** 10.1371/journal.pone.0110325

**Published:** 2014-10-15

**Authors:** Martin Stevens, Alice E. Lown, Alexander M. Denton

**Affiliations:** Centre for Ecology and Conservation, College of Life and Environmental Sciences, University of Exeter, Penryn Campus, Penryn, Cornwall, United Kingdom; National University of Singapore, United States of America

## Abstract

Camouflage is found in a wide range of species living in numerous habitat types, offering protection from visually guided predators. This includes many species from the intertidal zone, which must cope with background types diverse in appearance and with multiple predator groups foraging at high and low tide. Many animals are capable of either relatively slow (hours, days, weeks) or rapid (seconds and minutes) colour change in order to better resemble the background against which they are found, but most work has been restricted to a few species or taxa. It is often suggested that many small intertidal fish are capable of colour change for camouflage, yet little experimental work has addressed this. Here, we test rock gobies (*Gobius paganellus*) for colour change abilities, and whether they can tune their appearance to match the background. In two experiments, we place gobies on backgrounds of different brightness (black or white), and of different colours (red and blue) and use digital image analysis and modelling of predator (avian) vision to quantify colour and luminance (perceived lightness) changes and camouflage. We find that gobies are capable of rapid colour change (occurring within one minute), and that they can change their luminance on lighter or darker backgrounds. When presented on backgrounds of different colours, gobies also change their colour (hue and saturation) while keeping luminance the same. These changes lead to predicted improvements in camouflage match to the background. Our study shows that small rockpool fish are capable of rapid visual change for concealment, and that this may be an important mechanism in many species to avoid predation, especially in complex heterogeneous environments.

## Introduction

Predator-prey interactions have played a substantial role in shaping the diversity of life, leading to many adaptations and counter-adaptations for attack and defence [1]–[3]. Perhaps the most widespread defence is camouflage, preventing an object from being detected or recognised by an observer [4]. On a basic level, camouflage is intuitively simple, often involving matching the general appearance of the background environment. Yet this ignores a rich complexity to the subject because many different types of camouflage are thought to exist, from background matching and disruptive coloration to countershading and masquerade (e.g. [5]–[7]), and camouflage can also be optimised in a variety of ways.

Almost certainly, the most common form of camouflage in nature, and the basis for many other types of concealment, is background matching, where an animal resembles the general colour and pattern of the background [4], [8]. In nature, animals could exhibit background matching through a variety of mechanisms, including genetic adaptation over long time periods, phenotypic plasticity during development, behavioural choice of substrates, using materials and decorations from the environment, and colour change over different time scales (e.g. [9]–[13]). The ability to change colour over a short time frame, physiological colour change, is based on the redistribution of pigment within chromatophore cells [14]. Investigating animals that can change colour is a particularly useful method to study how camouflage works and is tuned to different environments because researchers can manipulate the background on which individuals are found and investigate how the camouflage is changed in response (e.g. [9], [15]). This approach can also yield insights into how the visual systems of animals work and interpret information in the environment, and how action is mediated via visual pathways [16].

While a great deal of recent research has investigated the different types of camouflage that may exist and how they work, mostly in artificial systems (e.g. [5], [6], [17]–[19]), comparatively little work has studied camouflage optimisation and tuning in real animals. The most extensive work to date has focussed on cephalopods, which show remarkable abilities for rapid colour and pattern change in response to specific features of the environment, such as pattern contrast and edge information [9], [16]. Studies of colour change for camouflage have also been undertaken in, for example, chameleons, flatfish, and crabs (e.g. [15], [20]–[22]). However, outside of studies on cephalopods, relatively little work has directly quantified how effective rapid (here defined as changes occurring in seconds or minutes) colour change for camouflage is, and how quickly this can occur. Little work has also directly investigated the exact form that colour change takes in terms of changes in colour and brightness. A major problem has been that conventional methods using spectrometry to quantify coloration require extensive handling of specimens, leading to stress induced colour change, and are also slow and hence unable to quantify rapid colour change.

Fish make an ideal group to study colour change for camouflage because it is widely reported that many species have this ability [23], and they occur in a wide range of habitats and on many backgrounds. The ability to change colour over short term, often for concealment, is thought to be widespread among teleost fish, both in the marine and freshwater environments [24], [25], and enables them to occupy a greater range of backgrounds and to cope with heterogeneous habitats. A variety of goby species have been observed to change colour, both to match their backgrounds in order to maintain camouflage [26], and during breeding phases [27]. However, only one study by Fries in 1942 [26] has specifically conducted experiments investigating camouflage by these fish on different backgrounds, with gobies reported to become paler and less red when on blue colours, darker and more red on red backgrounds, and more yellow on yellow backgrounds, owing to changes in chromatophore cells. However, colour change was monitored by human eye, without quantifying it objectively, how it affected match to the background, or how fast it occurred.

In this paper we study the colour change abilities of the abundant and widely distributed rock goby (*Gobius paganellus*) when placed on backgrounds of different colours and brightness, to test whether they can change appearance for camouflage, and how quickly they do this. We use digital image analysis and predator vision modelling to quantify the speed and extent of colour change. Rockpool and intertidal fish such as gobies are excellent candidate species for this type of study for a variety of reasons. First, the environment in which they live is highly changeable, with a wide range of background types existing even over very small areas. In addition, physical disturbance of tides and waves will often push individuals over a range of backgrounds against which they are viewed by predators. Furthermore, owing to the tidal nature of the environment, the fish are under intense predation pressure, at low tide from birds and at high tide from other groups, including larger fish. Therefore, there may be a major advantage in being able to rapidly change colour for camouflage.

## Materials and Methods

Gobies were collected by dip net in the intertidal zone from Gyllyngvase beach, Falmouth, Cornwall, UK (50° 8′33.4690″N, −005° 04′07.9716″W) between July 2013 and September 2013 for experiment 1 (40 individuals), and between October 2013 and July 2014 (40 individuals) for experiment 2. Once caught, fish were kept in fresh seawater in a grey bucket in order to minimise colour change prior to use. Both experiments were conducted *in* *situ* on Gyllyngvase beach under natural light conditions in shallow trays lined with waterproof paper (see below). All work was conducted under approval from the University of Exeter Biosciences ethics committee (application 2013/149). The field location (specified above) where the experiments were conducted and fish collected is public land and no further licences or permits were needed. Fish were kept no longer than 2 hours, and all individuals were returned unharmed to their original rockpool area after being tested. Rock gobies are not an endangered or protected species.

### Experimental Background Creation

Our aim in experiment 1 was to test whether gobies are capable of changes in their luminance when placed on a black or white background. In experiment 2, we aimed to test for changes in colour. Our design here was intended to minimise perceived differences in brightness of the backgrounds by the fish, and to test whether gobies change their actual colours as opposed to just changes in luminance. We used red and blue as two colours at different ends of the visual spectrum that gobies are likely to be able to discriminate (see Discussion). In both experiments we also aimed to test how quickly any changes in appearance occurred. Background colours for both experiments were created by printing colours at 300 dpi on waterproof paper (HP LaserJet Tough paper; Hewlett Packard, Palo Alto, USA), with a Hewlett Packard Colour LaserJet 2605 dn printer. Before the experiments, gobies were placed on an intermediate grey background for at least 15 minutes as a standard background for all individuals. This was to provide the same starting point at the beginning of the experiment, and to remove some of the individual variation that would otherwise exist owing to individuals being found on different rockpool substrates on collection. To produce an intermediate grey midway between white and black we followed past approaches [15] and printed a range of grey squares of different intensity (pixel) values from black through to white made in Photoshop Elements 5.0 (Adobe Systems Inc., San Jose, USA). We measured the reflectance of each square using an Ocean Optics (Dunedin, FL, USA) USB2000+ spectrometer, held at 45° to normal, with illumination by a PX-2 pulsed xenon lamp, and calculated the average reflectance of each square across 400–750 nm (we excluded UV light because the paper and the print toner reflect little UV light), followed by plotting image pixel value against reflectance. We then calculated the mid grey value based on a ratio scale [28]. For experiment 1, we simply printed the darkest black and used the white paper for the two experimental backgrounds. For experiment 2, the red and blue colours were calculated to be the same average brightness across the visible spectrum [15]. This was achieved by photographing a range of different red and blue colours printed on the same paper (as above), followed by measuring their reflectance values in longwave (LW), mediumwave (MW), and shortwave (SW) images.

### Experimental Procedure

Fish were placed in a 24 cm wide × 34 cm long × 5 cm deep (internal measurements) white tray that had been covered with a background of midpoint grey paper, calibrated as described above. The tray was filled 2 cm deep with fresh seawater and a spirit level was used to ensure trays were flat and the water level accurate to prevent variation in colour measurements due to water depth, and to ensure all areas of the tray had sufficient water to minimise stress to the fish by ensuring that even the largest individuals were fully submerged, while at the same time keeping water depth low so as to not affect the colour analyses. Fish were given 15 minutes to acclimatize on the grey background then photographed (see below) in the control tray before being transferred individually into a secondary experimental tray of 28.5 cm wide × 39 cm long × 7 cm deep (internal measurements) divided into eight compartments by thin plastic barriers attached with silicone sealant glue. These compartments were either four white and four black for experiment 1, or four red and four blue for experiment 2. Transfer of the fish between trays was done as quickly as possible and with a net in order to minimise any stress associated with capture and handling. Fish were unable to see each other, although barriers were not completely sealed and water was able to flow around the tray. Although this also meant that chemical cues could potentially transfer among individuals, any such effects should not produce directional colour changes in line with responses to background colours and brightness. The experimental trays also ensured that pairs of fish were tested under the same water conditions (e.g. temperature), and the relatively small size of the compartments prevented fish from swimming around too much, which would have made photography difficult.

Experimental trials were undertaken in blocks, with a single block consisting of a pair of fish, with one fish placed on each background colour, and with those individuals approximately matched by size to remove bias that may occur due to variation in colour change with individual size. Twenty fish were tested on each background colour for each experiment (40 fish in total per experiment). Fish were subsequently photographed again at 1–2, 10 and 60 minutes while remaining in the tray to establish the extent of colour change over time. Photos were taken using a Nikon D90 SLR camera, which had undergone a quartz conversion to enable ultraviolet sensitivity (Advanced Camera Services, Norfolk, UK) and fitted with a Nikon 105 mm Nikkor lens. In both experiments photographs were taken in human visible (400–700 nm) and ultraviolet (300–400 nm). For the human visible photos a UV/IR blocking filter was used (Baader UV/IR 2″ Cut Filter) and a UV pass filter was used during the ultraviolet photographs (Baader U 2″ Cut Filter). All photographs included a Spectralon 40% grey reflectance standard (Labsphere, Congleton, UK) next to the tray and a ruler. Due to changing light conditions and reflectance from the water surface, a black and silver photographic umbrella (Neewer, Guangdong, China) was used to shade the trays from direct sunlight.

### Image Analysis

Images were taken in RAW format with manual white balance and fixed aperture settings. Images were then linearized with regards to light intensity based on camera responses to a set of eight Spectralon grey standards with reflectance values ranging from 2 to 99% (in custom programs written in Image J) in order to correct for the non-linear responses in image values many cameras produce in response to changes in light levels [29]. Image values were then equalised with regards to the 40% grey standard, and each image channel (LW, MW, SW and UV) scaled to reflectance, where 255 on an 8-bit scale is equal to 100% reflectance [29].

We wanted to analyse colour change with regards to one of the likely main predator groups of rockpool fish: shore birds. To obtain data corresponding to avian vision, we transformed the reflectance based image based on spectral sensitivity data from the peafowl (*Pavo cristatus*) [30] using a polynomial mapping technique to convert from camera to avian colour space [15], [29], [31], [32]. The likely predators of rockpool fish include a range of shorebird species found at the intertidal zone. Previous work has shown that these are likely to have a ‘violet’ sensitive system [33], with the UV cone type shifted in sensitivity to slightly longer wavelengths than species that fall into the ‘ultraviolet’ group (although violet sensitive species can still detect UV light). Although gulls are likely to be predators of rockool fish too, and seem to have a UV visual system [33], the relatively low levels of UV involved in the backgrounds and fish should mean that differences in the perception between these systems is small. The peafowl is often used as a model species for modelling birds that fall into the violet group.

Once calibrated, the outline of each goby was drawn around by hand using Image J and the region of interest (ROI) saved. Each image layer was measured to acquire values for photon catch. We then calculated a series of metrics to analyse the appearance of each goby. Saturation (the amount of a given colour compared to white light) was defined as the distance an object is in a tetrahedral colour space from the achromatic grey point [34]. Larger distances equate to colours that appear more saturated. We next derived a measure of colour type, or hue. Here, we followed past approaches that have defined hue based on a ratio of the relative photoreceptor stimulation in different parts of the light spectrum [15], [35], [36]. Broadly, this approach, whereby colour types are defined in terms of a ratio of the different channels present, is based on the way that opponent colour channels are thought to work in animal vision, and in practical terms is a way of defining a colour type in an intuitive and readily interpretable manner [15]. In experiment 1, we had no *a priori* reason to expect particular changes in colour of fish because all the backgrounds used were achromatic shades of grey. Therefore, we used a standardised ratio that describes colour in terms of differences in the amount of shorter to longer wavelengths of light (an approach commonly used to calculate opponent channels): hue = ((LW+MW)–(SW+UV))/(LW+MW+SW+UV). In experiment 2, whereby we used backgrounds that were either red or blue, we predicted specific changes in coloration with fish moving more towards these two colour types. As such, we defined hue as (LW–SW)/(LW+SW). Higher values mean that an individual is relatively red in colour, whereas smaller values mean an individual is relatively blue. To derive a measure of achromatic change in appearance, we calculated luminance (perceived lightness) based on the double cone values, as in birds achromatic vision is widely thought to be driven by these receptors [37].

Finally, we calculated how changes in the appearance of fish equated to differences in their level of match to the experimental backgrounds. To do so we used a log form of a model of visual discrimination, the Vorobyev-Osorio model [38], which is based on differences in colour or luminance based on photo catch values, including estimates of neural noise and relative photoreceptor proportions. We used a Weber fraction value of 0.05 for the most abundant cone type [70, 90], and relative proportions of cone types in the retina of the peafowl (LW = 0.95, MW = 1.00, SW = 0.86, UV = 0.45; [30]). The model gives values of ‘just noticeable differences’ (JNDs), whereby differences of 1.00–3.00 mean that two stimuli are unlikely to be discriminated by an observer, and larger values above 3.00 are increasingly likely to equate to discriminable differences [39].

### Statistics

We did not specifically expect an overall difference in appearance between fish on each background at all time points. Instead, our key prediction was that there should be no difference at the start of the experiment (time zero) when fish have been on the same intermediate grey background, whereas there should be differences as the experiment progresses. The exact time where differences arise should also depend on the speed of colour change. As such, we conduced a series of planned comparisons [40] between fish on each background type, separately at each time point. Data for all metrics except hue were non-normal and resistant to transformation and so we conduced Wilcoxon Mann-Whitney tests. For hue we conducted two-sample t-tests. Owing to the repeated testing for each experiment (one test per time point), we adjusted the critical p-values needed for significance by using a sequential Bonferroni [41]. For each experiment, p-values are ranked in order of significance and then compared to an adjusted critical value in turn, which becomes more stringent with each additional test. Critical thresholds for significance for each of the four statistical tests per experiment were therefore 0.050, 0.025, 0.016, and 0.012. To test for changes in the level of camouflage over time for both colour and brightness/luminance, we conducted Kruskal-Wallis tests.

## Results

### Experiment 1

#### Changes in Colour and Luminance

**Figure 1:**
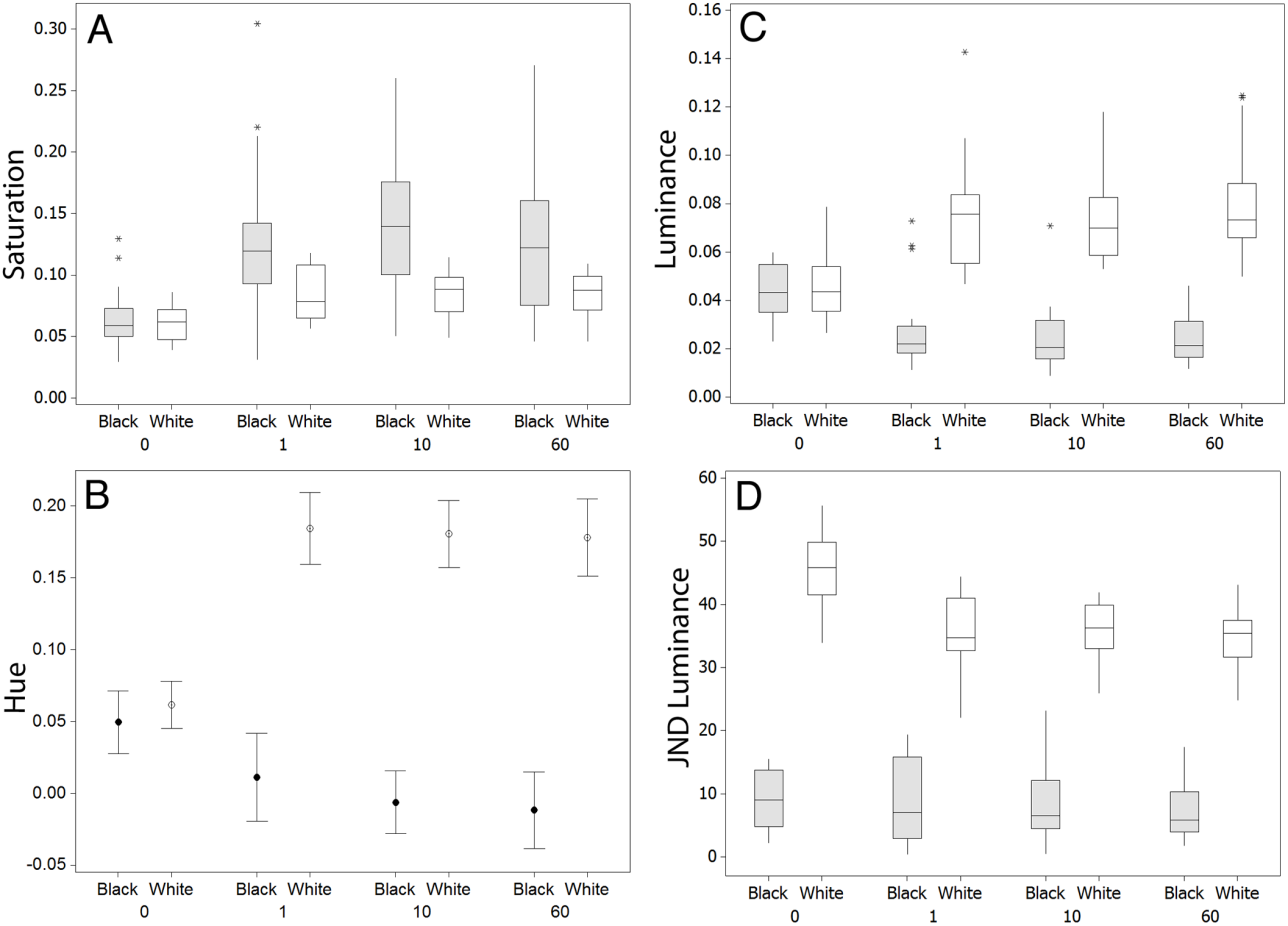
Changes in the different colour and achromatic metrics for fish on black and white backgrounds in experiment 1 for saturation (A), hue (B), and luminance (C) at the start (0 minutes) of the experiment and at 1, 10, and 60 minutes. Panel D shows the level of similarity for fish against the white and black backgrounds for JNDs in luminance (see main text) at 0, 1, 10, and 60 minutes. Graphs A, C, and D show medians plus inter-quartile range (IQR), whiskers are lowest and highest values that are within 1.5*IQR from the upper and lower quartiles, asterisks represent outliers. B shows means plus standard error.

For luminance, there was no significant difference between fish on black or white backgrounds at time 0 (W = 414.0, n = 20, p = 0.925), but there were significant differences at one minute (W = 587.0, n = 20, p<0.001), 10 minutes (W = 600.0, n = 20, p<0.001), and at 60 minutes (W = 610.0, n = 20, p<0.001), with fish on white backgrounds having higher luminance values (figure 1). Note, however, that the magnitude of differences is generally quite small with the largest difference in luminance values between time 0 and time 60 being 0.09 (with photon catch values on a scale of 0–1), and average differences being 0.03 (across both backgrounds).

In terms of colour change, for saturation, there was also no significant difference between fish on black or white backgrounds at time 0 (W = 406.0, n = 20, p = 0.925), but significant differences occurred at one minute (W = 305.0, n = 20, p = 0.005), 10 minutes (W = 268.0, n = 20, p<0.001), and 60 minutes (W = 308.0, n = 20, p = 0.006), with saturation values being higher on the black backgrounds (figure 1). The results for hue were very similar, again with no significant difference at time 0 (T = −0.92, df = 35, p = 0.363), but significant differences at one minute (T = −9.15, df = 36, p<0.001), 10 minutes (T = −12.24, df = 37, p<0.001), and 60 minutes (T = −10.49, df = 37, p<0.001), with hue values being higher (more LW and MW and less SW and UV in colour) for fish on white backgrounds (figure 1).

#### Differences in Camouflage Over Time

On the white background there was no significant reduction in JNDs over time (better match to the substrate) for colour (H = 0.46, df = 3, p<0.927), but there was for luminance JNDs (H = 31.77, df = 3, p<0.001; figure 1D). On the black background there was a significant difference in colour JNDs with time (H = 12.81, df = 3, p = 0.005). However, note that there was no decline in JNDs with time, but rather lower JND values at times 0 and 60 than at times 1 and 10, indicating that fish did not actually improve camouflage for colour over time intervals. There was no significant difference in luminance JNDs (H = 1.59, df = 3, p = 0.661). Therefore, fish improved in their achromatic match to the white background, but not to the black background.

### Experiment 2

#### Changes in Colour and Luminance

**Figure 2:**
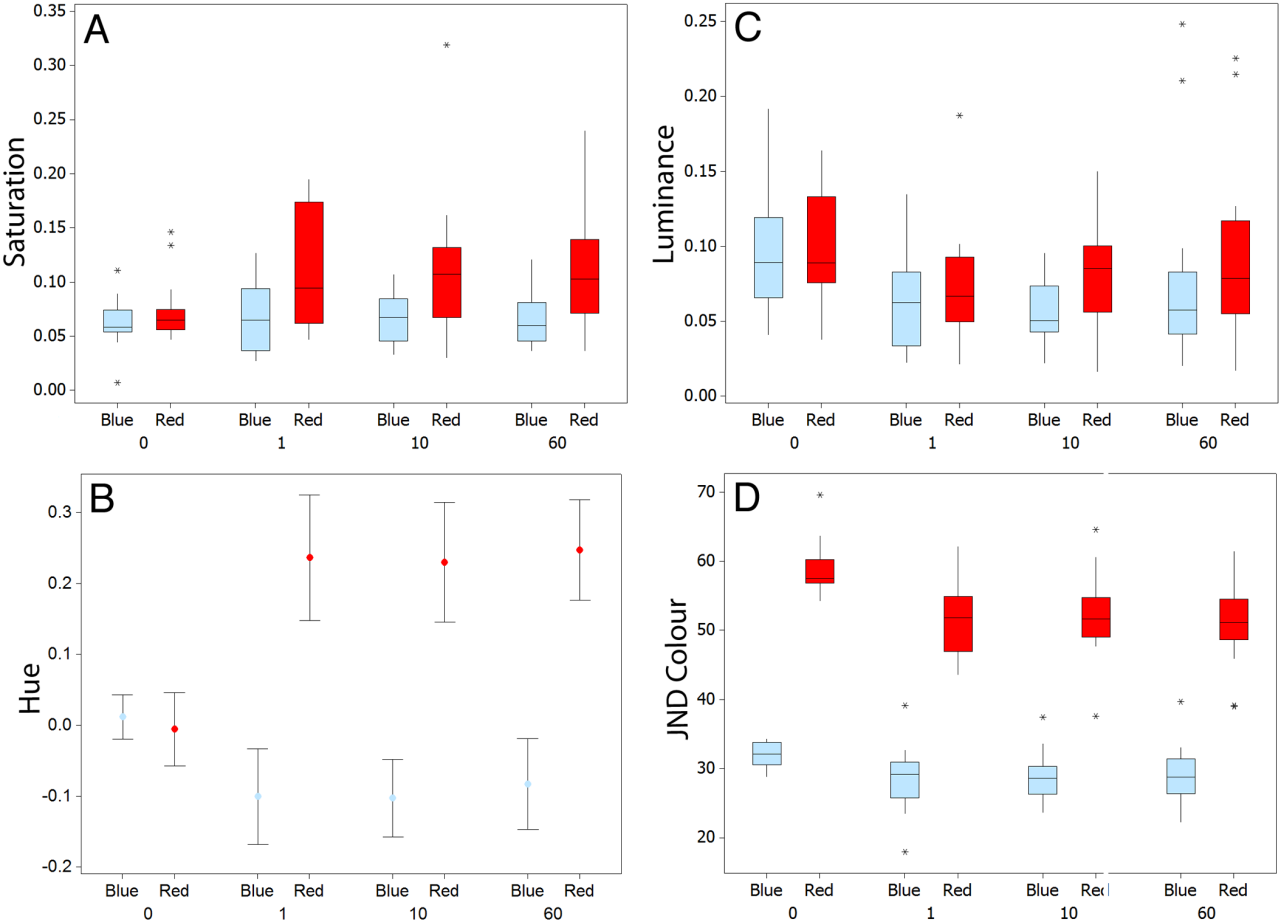
Changes in the different colour and achromatic metrics for fish on red and blue backgrounds in experiment 2 for saturation (A), hue (B), and luminance (C) at the start (0 minutes) of the experiment and at 1, 10, and 60 minutes. Panel D shows the level of similarity for fish against the red and blue backgrounds for JNDs in colour at 0, 1, 10, and 60 minutes. Graphs A, C, and D show medians plus inter-quartile range (IQR), whiskers are lowest and highest values that are within 1.5*IQR from the upper and lower quartiles, asterisks represent outliers. B shows means plus standard error.

There was no significant difference between fish on red or blue backgrounds for luminance at time 0 (W = 410.0, n = 20, p = 1.000), at one minute (W = 373.0, n = 20, p = 0.324), or at 60 minutes (W = 346.0, n = 20, p = 0.086), nor was there a significant difference at 10 minutes when controlling for multiple testing (W = 327.0, n = 20, p = 0.025); figure 2.

**Figure 3:**
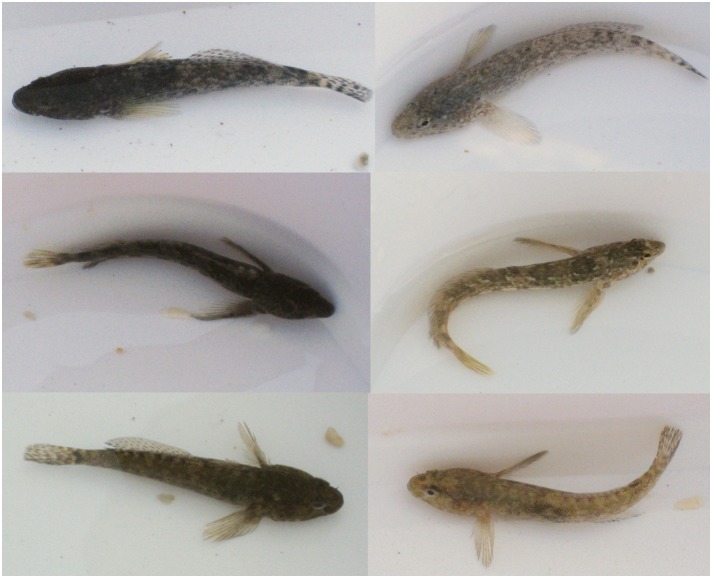
Examples of changes in brightness of fish. Three individuals are shown on the left having been placed on a black background, and then the same individuals are shown on the right after being on a white background.

Regarding colour, for saturation, there was no significant difference between fish on red or blue backgrounds at time 0 (W = 378.0, n = 20, p = 0.394), but there were significant differences at one minute (W = 311.0, n = 20, p = 0.008), 10 minutes (W = 307.0, n = 20, p = 0.006), and 60 minutes (W = 289.0, n = 20, p = 0.001), with fish being more saturated on the red background (figure 2). The results for hue were similar, with no significant difference at time 0 (T = 0.60, df = 31, p = 0.552), but significant differences at one minute (T = −6.35, df = 35, p<0.001), 10 minutes (T = −6.90, df = 32, p<0.001), and 60 minutes (T = −7.24, df = 37, p<0.001). Fish on red backgrounds had higher hue values (more LW and less SW in coloration; figure 3).

#### Differences in Camouflage Over Time

On a red background, there was a significant reduction in JNDs for colour (H = 25.31, df = 3, p<0.001), but not for luminance JNDs (H = 3.10, df = 3, p = 0.376); figure 2D. On a blue background, there was also a significant reduction in colour JNDs over time for colour (H = 17.56, df = 3, p = 0.001). Although there was a significant change in luminance JNDs, this was generally a decrease rather than improvement in luminance matching over time 0 (H = 14.64, df = 3, p = 0.002).

## Discussion

Here, we tested whether rock gobies can change either their luminance (lightness) or colour depending on the background on which they are placed. As predicted, in experiment 1, fish changed in their overall luminance when put onto either a white or a black background, with individuals getting lighter or darker respectively. This led to changes in the level of similarity of fish to each background in terms of luminance, improving camouflage matching over time. In contrast, although there were some statistically significant changes in hue and saturation in this experiment too, these generally did not affect the overall match to the background, indicating that these changes were perceptually small and unlikely to be of significance in terms of camouflage, similar to other work [15].

In contrast, in experiment 2 where fish were placed onto either red or blue backgrounds, individuals underwent marked changes in colour with regards to both hue and saturation. At least some goby species have been shown to have three cone types, sensitive to relatively shorter and medium/longer parts of the spectrum [42], and so they should be able to distinguish between the blue and red backgrounds. In accordance with this, on a red background fish became more red in colour and more saturated, whereas on a blue background they became less red more grey in colour. These differences led to significant improvements in the level of colour match to the background. In contrast, there was little change in the luminance of fish on these backgrounds, demonstrating that fish can change their overall colour without changing their luminance. The exact mechanism of luminance perception in rock gobies is unknown, but this result suggests that they likely perceived the two background types as being of about the same brightness because in experiment 1, where the backgrounds were very different, fish did change in luminance. Overall, in both experiment 1 and 2 the changes were very rapid, with the majority of colour and brightness change occurring in the first minute.

The result that fish changed to become more red in coloration on the red background, yet that changes towards the blue colour were much smaller (they mostly become more grey in colour) is interesting. It suggests that some types of colour are easier for the fish to adopt than others. This fits with the background environment in the habitat where the fish were collected, whereby blue colours are rare, yet red encrusting algae and brown stones and seaweed are common. Past work has shown that different types of chromatophore control different colours, with black melanin being controlled by melanophores, yellow pteridine controlled by xantophores, red carotenoids controlled by erythrophores and more rarely blue cyanophores controlling a yet unknown cyan biochrome [25], [26]. Colour responses may also be elicited by more than one type of chromatophore, and Fries [26] suggested that blue response in common gobies are due to a response of the erythrophores, xanohpores, and iridophores. However, more work is needed to test what cellular mechanisms cause changes in goby coloration, and whether other populations might be capable of greater changes in blue.

The levels of change in luminance were relatively small in this study even for the fish that changed the most. Thus the decrease in difference to the background, although significant, was not very large. However, this did equate to a decrease in discrimination thresholds of almost 10 JNDs on average for fish on the white backgrounds. In general across the experiment fish were quite dark, and in nature they are likely to be a better match to the general colour and brightness of the substrate in the rockpools (especially the dark rocks). Therefore, in such cases in the wild when fish are already well matched in appearance to the background even relatively small differences may equate to a valuable benefit in improved camouflage. Furthermore, to our eyes, changes in the brightness of fish are clearly perceptible (figure 3). One possibility to resolve this apparent discrepancy is that in this study we analysed the appearance of the entire body of each fish. In reality, gobies often have quite strong patterns that to us have key characteristics of disruptive coloration to break up the body shape against the background [1], [4], [5]. We often noted that the prominence of such patterns changed as fish change colour, and we think it quite possible that even when the overall brightness of an individual stays essentially the same that there can be pronounced changes in pattern. For example, on a uniform background fish may reduce the contrast and prominence of their markings and adopt a more uniform appearance, but this may be broadly similar in overall brightness to that of their starting appearance. Otherwise, better brightness match to the background may be brought about through longer-term changes, such as through morphological colour change that occurs over days and months and may be caused by changes in the overall density of chromatophores in the skin [14].

It is interesting that the ability of fish to change colour seems to be better than their ability to change brightness. Until we test fish on more natural coloured backgrounds we can only speculate as to why this may be. In the rockpool environment, the background is highly heterogeneous in terms of brightness, with stones and gravel of a range of shades occurring on a small scale (smaller than the size of the fish). Thus, overall changes in brightness may have a relatively small benefit. In contrast, some rockpools and larger backgrounds seem to have broadly different colours, meaning that colour change may be more valuable. This is likely to be especially the case with changes in shore height too, whereby there are changes in the amount of substrate types, especially greater brown and green algae cover lower down the shore.

Here, we have focussed on changes in colour and brightness using relatively artificial background appearances. Next, it will be important to test for colour change and camouflage ability on backgrounds that more closely resemble those in the environment where the individuals live. Moreover, given that gobies often have strongly contrasting patterns that appear disruptive, it would be important to test whether individuals have the capacity to change their markings on backgrounds of different marking sizes and contrasts. Previous studies have shown that flatfish are capable of impressive changes in pattern depending on the substrate appearance [20]. While gobies are unlikely to match the extent of this ability, the potential is there for them to change their patterns for concealment. In addition, the environment in which gobies live is both highly challenging (the intertidal) and heterogeneous, and so being able to adjust individual markings is likely to provide a strong advantage. A number of other species live in the same habitat, including several other common species of goby and blenny that have been suggested to change colour too. Thus, there also exists great potential for comparative studies of colour change within and among habitat types in intertidal fish species. It should also be noted that in this study we have not directly explored the level of individual colour change possible because this would require placing the same individual fish on different backgrounds in a repeated measures design and analysing their colour change abilities.

While in the present study we have focussed on colour change for camouflage a number of recent studies on colour change in gobies report change colour in response to breeding, with individuals becoming less camouflaged and more attractive to mates [43], [44]. This change appears to be largely hormonally induced [44]. In gobies, breeding coloration change in other species is also influenced by predation pressure, with courtship colouration less intense under high predation risk [45], [46]. When in aquaria devoid of predators, however, blennies from high predation sites showed full courtship colouration [46]. In other fish, arctic charr (*Salvelinus alpinus*) have shown differences in aggression when paired over white or black backgrounds [47], [48]. Darker skin colour in salmonids may relate to subordination and be used to reduce aggressive interactions between conspecifics. When fish are paired within a tank and acclimatised to light coloured backgrounds, fish generally show increased aggression towards each other, whereas this aggression is not observed if fish have been acclimatised to dark backgrounds [47]. Many species of fish will also change colour under stressful conditions, such as when odour cues suggest a predator is in close proximity [49]. While some species of goby have been found to react to this predator cue, *Gobius paganellus* has not been found to display any defensive reaction to conspecific skin extract ([49] – see [50]). In *Gobius minutus*, physical handling also provoked rapid darkening and reddening in pale fish [26]. Thus, it would be valuable to investigate how capacity for colour change is affected by factors such as season and mating behaviour, dominance, and condition. Overall, colour change in fish and other species presents an excellent system to study camouflage, what makes this effective and how it is controlled, and other life history factors that may affect camouflage responses and tuning.

## Supporting Information

Data S1 **Experimental data.**
(XLSX)
